# A Case of Extra-Uterine Pregnancy

**Published:** 1849-02

**Authors:** W. W. Harbert

**Affiliations:** Shelby county, Kentucky


Art. III.—*A Case of Extra-Uterine Pregnancy.* By Dr. W. W. Harbert, of Shelby county, Kentucky.

Jane, a black girl, belonging to Elijah Yager, of Shelby county, Kentucky, aged 23 years, of good constitution, and the mother of a living child, in the sixth month of her second pregnancy, on the morning of the 21st January, 1848, complained of slight indisposition. Notwithstanding, she went to the field to attend to some business, but in a short time returned, complaining of being much worse. I was immediately summoned; and upon my arrival found her strongly threatened with abortion. She complained of a feeling of weight and tension across her loins, with occasional uterine contractions and concomitant pains. Pulse slightly accelerated, but of small volume. She was sitting up. I ordered her an anodyne, a full dose of castor oil, warm pediluvia, etc.

22d. The urgency of the symptoms somewhat abated; the oil operated freely; pulse continues too frequent and is more easily compressed. I ordered the anodyne to be repeated; enjoined quietude in the recumbent position, and a light unirritating diet.

23d. The patient expressed herself as feeling much better; was cheerful, and thought that she would soon be well.

24th. Patient still apparently better; seems rather dull and stupid, but complains of no particular pain. Skin dry and rather cool; pulse contracted and rapid. No medicine.

25th. Patient apparently improving. No medicine.

26th. Patient still up, and able to go to the barn to attend to some business, but had not been there long when she suddenly grew faint and sick, and commenced vomiting. She was assisted to the cabin, and I was immediately requested to see her. In an hour from the occurrence of the symptoms above related, I arrived, and found her complaining very much of a sense of weight and pain in the right side, indeed all the former symptoms much aggravated. Pulse rapid, 115 in the minute; continued nausea. She complains also of very distressing pain, and a sense of tightness in the hypogastric region. Extremities cold; cramp in the legs; surface of the chest and abdomen dry and hot; thirst urgent. The motions of the fœtus were distinctly felt by laying the hand on the abdomen; a vaginal examination discovered a hot and rigid condition of the os uteri, with no dilatation. There was a sanious discharge from the vagina. I ventured on the abstraction of twelve ounces of blood; exhibited nitric ether; ordered tepid or hot pediluvia; and an aperient, to be followed by an anodyne at night.

27th. The patient rested badly through the night.

Bowels had been slightly moved; pulse small and very rapid; abdomen considerably enlarged, tense and hot. I ordered an aperient and fomentations. No dilatation of the mouth of the womb.

28th. The aperient has not acted, and the patient is much worse. Dr. E. Bryan was requested to see her in consultation. We found the abdomen greatly swollen and tense; bowels constipated; pulse 130. Fomentations continued; ordered a large dose ol. ricini and oil turpentine.

Evening. All the symptoms worse; bowels have not been moved. Exhibited three drops of croton oil, to be followed by enemata.

29th. Bowels not moved; patient complains of a distressing sense of suffocation; abdomen more distended; pulse 130. Oil and turpentine, with the enemata, to be repeated.

30th. No alvine dejections; pulse remarkably quick; extremities cold; abdomen still larger and more tense; urgent thirst: in short, all the symptoms of approaching dissolution present. She expired at midnight.

*Autopsy* was made nine hours after death. My friend, Dr. Freeman, who was invited to be present, kindly conducted the examination, in the presence of Dr. Bryan and myself. On opening the abdomen, the following appearances were presented: A clot of blood, of the size of a large placenta, lay in the hypogastric region, in front of the abdominal viscera. The ovum, with the membranes entire, was found ex utero, in the peritoneal cavity, lying to the right side of the mother, under the omentum. The placenta was attached to the fundus uteri, in or to a cup-like development of that part of the viscus. The fœtus, funis umbilicalis, and placenta, were of the size they usually are at the sixth month of utero-gestation. The peritoneal cavity was full of a bloody serum. The body and neck of the uterus were but little enlarged—not larger than a large lemon, aside from the cup-shaped development for the attachment of the placenta. On laying open the cavity of the uterus the membrana decidua was plainly seen in a partially separated state. The left Fallopian tube entered the cavity of the uterus much lower down than the right; indeed I was not able to trace the entrance of the right tube into the uterine cavity. I could not detect a corpus luteum in either of the ovaria. By reason of an acute ophthalmia, Dr. Freeman was incapacitated for a minute examination.

The cause of death was evidently internal hemorrhage, from a partial rupture of the utero-placental connection. Whether the Cæsarean operation would promise anything in similar cases, were the diagnosis made in time, is an inquiry that can only be answered by observation and experience.

December 20, 1848.

